# Multilamellar hyaluronic acid-*b*-poly(lactic acid) polymersomes for pathology-responsive MRI enhancement†

**DOI:** 10.1039/d4bm01583e

**Published:** 2025-04-17

**Authors:** Dorian Foster, Naisha Shah, Alaura Cakley, Ronald Beyers, Jessica Larsen

**Affiliations:** a Department of Chemical and Biomolecular Engineering, Clemson University Clemson SC 29634 USA larsenj@clemson.edu; b South Carolina Governor's School for Science and Mathematics Hartsville SC 29550 USA; c Auburn University MRI Research Center, Department of Electrical and Computer Engineering, Auburn University Auburn AL 36849 USA; d Department of Bioengineering, Clemson University Clemson SC 29634 USA

## Abstract

This study introduces a biocompatible, stimuli-responsive imaging and therapeutic delivery system using ultrasmall iron oxide nanoparticles (USPIONs) encapsulated within the hyaluronic acid-*b*-poly(lactic acid) (HA–PLA) polymersome membrane, with a model protein bovine serum albumin in the core. These multilamellar vesicles exhibit enhanced *T*_2_-weighted MRI contrast, achieving a relaxivity 3-fold higher than existing agents. The polymersomes demonstrate acid- and enzyme-triggered degradation, enabling controlled release and measurable contrast changes in pathological environments. Preliminary *in vivo* and postmortem studies confirm their strong imaging performance, high biocompatibility, and targeted response to enzymatic, acidic microenvironments, paving the way for theranostic applications in disease diagnosis and treatment monitoring.

## Abstract 2

This study introduces a biocompatible, stimuli-responsive theranostic system with ultrasmall iron oxide nanoparticles (USPIONs) encapsulated within the hyaluronic acid-*b*-poly(lactic acid) (HA–PLA) polymersome membrane and a model protein in the core.

## Graphical abstract

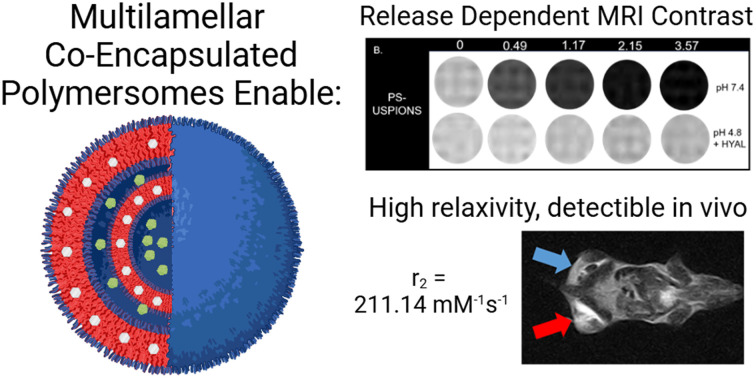

## Introduction

Imaging plays an integral role in diagnostics, disease and treatment monitoring. Magnetic resonance imaging (MRI), which generates two- and three-dimensional images using the natural magnetic properties of water content throughout the body, is a leading choice for soft tissue imaging.^1^ It is non-invasive and does not require non-ionizing radiation. Although it has high temporal and spatial resolution, MRI has low sensitivity. Contrast agents (CAs) are often necessary to compensate for this low sensitivity. Although other mechanisms exist, MRI CAs typically enhance contrast by altering the relaxation time of nearby water protons.^1^ Stimuli-responsive CAs can further improve the sensitivity and specificity of CA-enhanced MRI, as they experience a measurable change in relaxivity in response to a change in environment, usually one that is characteristic of pathology or organelle-specific physiology.

Some of the most common stimuli around which responsive systems are designed are pH and enzyme activity. Polymer functionalization can integrate stimuli-responsivity, improving CA performance upon pathologic exposure. Solubility-switching polymers have been applied to directly enhance CA-water interactions^2^ or cause CA aggregation.^3^ Polymeric encapsulation has also been used to shield CAs from aqueous exposure temporarily,^4^ muting contrast in neutral conditions. Here, we have developed an acid and enzyme-responsive iron oxide (IO) CA through polymersome (PS) encapsulation. Although IO CAs are not predisposed to stimuli-responsive activity, polymer functionalization has been able to induce tuneable, stimuli-triggered relaxivity changes.^1^ PSs, nanoparticle vesicles formed from the self-assembly of amphiphilic block co-polymers, can co-encapsulate both hydrophilic and hydrophobic drugs,^5^ which could enable the future addition of therapeutics alongside IO CAs.

Previously, our lab has developed highly biocompatible, stimuli-responsive PSs made from hyaluronic acid (HA) and polylactic acid (PLA).^6,7^ HA–PLA PSs degrade under acidic conditions and upon exposure to enzymes like hyaluronidase or hexosaminidase A, with quantitative release profiles changing in response to changing HA molecular weight and hyaluronidase concentration.^7^ Building on this, we co-encapsulated ultrasmall IO nanoparticles (USPIONs) into HA(5 kDa)–PLA(15 kDa) PSs with bovine serum albumin (BSA) as a model protein to enable nanoparticle tracking *via* MRI. Our work lays the foundation for theranostic PSs for simultaneous protein delivery and disease monitoring.

## Materials & methods

### Materials

Amine functionalized iron oxide (Fe_3_O_4_) nanoparticles (3 nm) were purchased from US Research Nanomaterials, Inc (Houston, TX) due to their contrast enhancement properties when used with MRI (Fig. S1†). Tween20 (Sigma Aldrich, MO, USA) was used as a surfactant to prevent USPION aggregation for size characterization. *N*-Hydroxysuccinimide (NHS) activated poly(lactic acid) (PLA-NHS, MW 15 kDa) and hyaluronic acid (HA, MW 5 kDa) were purchased from Creative PEGworks (Durham, NC. USA). Float-a-lyzer dialysis devices (encapsulation studies) and slide-a-lyzers (release studies), both of MWCO 100 kDa, came from Thermo Fisher Scientific (Waltham, MA, USA). Phosphotungstic acid (PTA, Polysciences, Inc, Warrington, PA, USA) was provided by the Clemson Electron Microscopy Facility for polymersome staining. Ferrozine (Cayman Chemical Company, MI, USA) was used in iron quantification assays. Fluorescein isothiocyanate (FITC) tagged BSA and hyaluronidase (HYAL) were purchased from Sigma Aldrich (St Louis, MO, USA). Agarose (VWR International, PA, USA) was used to suspend iron nanoparticles for MRI phantom imaging. HYAL was purchased from Sigma Aldrich (MO, USA).

SHSY-5Y cells were purchased from American Type Culture Collection (VA, USA) for *in vitro* studies. Media was 1 : 1 mixture of Eagle's Minimum Essential Medium and F12 Medium (Thermo Fisher Scientific, Waltham, MA, USA) supplemented with 10% Fetal Bovine Serum (FBS) (Sigma Aldrich, MO, USA) and 1× Penicillin–Streptomycin (Thermo Fisher Scientific, Waltham, MA, USA). Passages were performed using 0.05% Trypsin (Corning Inc., Corning, NY, USA). Cytotoxicity was evaluated using an MTS Cell Proliferation Assay kit from BioVision (San Francisco, CA, USA). βgal^−/−^ heterozygous mutant mice (Strain #:037063) were obtained from The Jackson Laboratory (Bar Harbor, ME, USA) for *in vivo* studies (AUP-2023-0215). Age-matched (five-month-old) mutant βgal^−/−^ mice and wild-type (WT) mice from our breeding colony were used for live imaging studies; three WT mice (6 months of age, 1 female, 2 male) were used in the *postmortem* imaging study (AUP-2023-0402). All chemicals and reagents were of analytical grade.

### Methods

#### USPION surface coating by PLA

USPIONs were coated with PLA(15 kDa), the same polymer as that of the hydrophobic block of the HA–PLA copolymer. By doing so, a polarity match was created between the USPIONs and the PS membrane in order to ensure membrane localization upon USPION encapsulation. Polymer coating occurred *via* a facile reaction of the amine on the pre-functionalized USPIONs and NHS group of the activated PLA(15 kDa)-NHS. The USPIONs and PLA-NHS were each separately dissolved in DMSO before mixing at a 20% by mass excess of PLA-NHS. The reaction vessel was then shaken at room temperature for 2 h. The conjugated USPIONs were isolated *via* centrifugation after which they were resuspended in Type 1 water and lyophilized to yield PLA-USPIONs as the final product. Size and surface charge characterizations by dynamic light scattering (DLS) (Zetasizer Nano ZS90 instrument, Malvern, UK) were used to confirm polymer coating. Tween20 (1% on a volume basis) was added as a surfactant to prevent aggregation of naked UPSIONs and PLA-USPIONs only during DLS readings to confirm PLA attachment. Because polymersomes stabilize USPIONs, Tween20 was not included in any PS formulations or measurements. TEM images were also taken as qualitative support for DLS results. TEM was performed on a Hitachi 7830 UHR (Tokyo, Japan) at 120 kV.

#### Polymersome assembly and loading

The HA–PLA copolymer was synthesized as described previously.^6,7^ USPION loading was achieved in HA–PLA PSs by integrating PLA-USPIONs or unconjugated USPIONs into the HA–PLA during a solvent injection protocol developed by our lab.^7,8^ USPIONs were first dissolved in DMSO. 2.4 mg of HA–PLA was dissolved into 100 μL DMSO or USPION/DMSO suspension with 0, 1, 5, 10, 15, or 20 μg USPIONs. Using a syringe pump, the organic phase mixture with HA–PLA and USPIONs was injected into a solution of 8 wt%/volume mannitol (used as a lyoprotectant^9^) in Type I deionized water at a rate of 20 μL min^−1^ using a 21-gauge needle. The PS solution was then filtered through a 0.45 μm syringe filter. PSs were characterized for hydrodynamic diameter (HD) and zeta potential (ZP) *via* DLS. This loading protocol was optimized for maximal USPION concentration by adjusting the percentage of PLA-USPION/USPION to DMSO. USPION loading was evaluated using a basic colorimetric ferrozine assay.^10,11^ TEM images were taken to confirm PS formation and USPION localization into the PS membrane. PSs were prepped for TEM with 1% PTA for negative staining.

PSs were co-loaded with FITC-BSA, serving as a model hydrophilic drug/protein. 10 μL of 2 mg mL^−1^ FITC-BSA solution was vortexed with 10 mg of lyophilized PSs; 990 μL of Type 1 deionized water was then added and mixed to obtain a total volume of 1 mL of the loaded PS solution. The loaded PSs were then added into a float-a-lyzer (MWCO 100 kDa) for dialysis against deionized water. Samples of the dialysate were taken hourly to identify FITC-BSA release over time. Each time a sample was taken, the buffer was changed to maintain a concentration gradient and encourage complete diffusion of the released protein into the dialysate. The total FITC-BSA released into the buffer (*C*_r_) was quantitatively analyzed by using UV-vis spectroscopy (BioteK Synergy H1); any FITC-BSA not released through dialysis was assumed to be encapsulated in the PSs. EE was calculated using the following equation:1
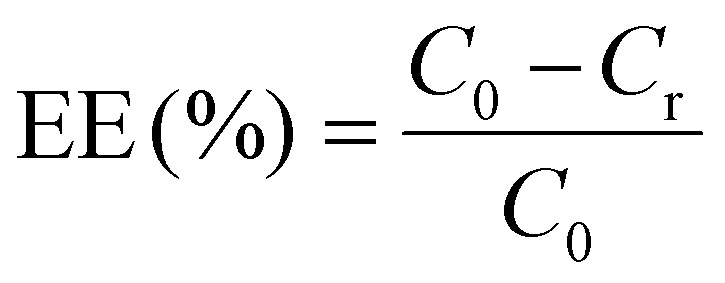
where *C*_0_ is the original concentration of FITC-BSA added to the lyophilized PSs prior to dialysis and *C*_r_ is the measured concentration as calculated from fluorescent signal.^7^

#### MRI characterization

Samples were suspended in phosphate-buffered saline (PBS) (pH of 7.0 or 4.8 + 1 mg mL^−1^ hyaluronidase) solution (PS-USPIONs) or in 2% agarose solution (USPIONs, PLA-USPIONs). In the timed-release study, only PS-USPIONs were examined but were prepared at various timings preceding a single *T*_2_ imaging sequence. Sample tubes were arranged as an array of rows/columns and submerged in a saline water bath tray (200 mL). MRI was acquired by a Siemens 3 Tesla SkyraFit using the human knee coil. For *T*_1_-weighted images, an inversion recovery experiment was performed with pulse sequence repetition time *T*_R_ = 10 s and inversion time *T*_I_ times [(ms): 10.0, 30.0, 50.0, 75.0, 100.0, 150.0, 200.0, 275.0, 350.0, 450.0, 550.0, 700.0, 850.0, 1050.0, 1250.0, 1500.0, 1750.0, 2000.0, 2300.0, 2600.0], generating 20 individual images. For *T*_2_-weighted images, 12 images were generated each with constant *T*_R_ and echo time (*T*_E_) as follows: *T*_R_ = 6.0 s and *T*_E_ (ms): 7.5, 20.0, 40.0, 70.0, 100.0, 150.0, 225.0, 300.0, 400.0, 550.0, 750.0, 1000.0. Images were analyzed with an in-house (Auburn University) written Matlab program to produce first-order exponential *T*_1_ and *T*_2_ recovery curves followed application of a Rosenbrock minimization method to estimate the *T*_1_/*T*_2_ and *R*_1_/*R*_2_ time constants. The *r*_2_ and *r*_1_ relaxivities were calculated according to the slope of the plot of *R*_1_ and *R*_2_ against Fe concentrations (Fig. S2 and S3†).

#### *In vitro* studies

Neuroblastoma cells isolated from humans (SHSY5Y cells) were seeded separately for 4-hour and 24-hour treatments and allowed to attach overnight preceding treatment. Following rinsing with 0.1 M PBS, cells were treated with USPIONs, PLA-USPIONs, or PS-USPIONs that had been suspended in fresh media at set concentrations; cells were incubated at 37 °C and under 5% CO_2_ for the entirety of the corresponding treatment window. Cytotoxicity was evaluated using an MTS assay as follows. At the end of the treatment period, MTS reagent was added to the media at 10% by volume of the total working volume of the well. The cells were incubated for another 4 hours before absorbance readings were taken at 490 nm on a UV-vis BioteK Synergy H1. Cell viability was quantified as a ratio of the absorbance of the treated cells to the absorbance of untreated control cells.

#### Postmortem imaging

All animal procedures were performed in accordance with the Guidelines for the Care and Use of Laboratory Animals and approved by the Institutional Animal Care and Use Committee of Clemson University with Animal Use Protocols 2023-0215 and 2022-0402. Three wild-type C57BL/6 mice (AUP-2022-0402) were sacrificed at 6 months (1 female, 2 male) then injected intramuscularly in both rear hind legs with either 500 μL of PS-USPION solution (3.2 μg Fe per mL in 225 mg mL^−1^ PS-USPION) or 500 μL of PBS. Immediately following injection, MRI was acquired by an Easote 0.25 Tesla Vet-MR GRANDE scanner using a knee coil. *T*_2_-weighted images were collected using two different MRI sequences – Spin echo (SE) and 3D steady state gradient echo (3D SSR2). SE images were generated with constant *T*_R_ and *T*_E_ of 20 ms and 10 ms, respectively. 3D SSR2 images were taken with a constant *T*_E_ of 120 ms, but the *T*_R_ setting was determined automatically by the Easote imaging software based on the number of equally sized slices required to image the entire mouse (*T*_R_ ranged from 2180–2970 ms). To account for variable *T*_R_, image quantification is normalized to the internal control of the PBS-injected leg. ImageJ was used to quantify relative integrated density values, normalized to the internal PBS-injected leg (Fig. S4†). Regions of interest (ROIs) were drawn around each leg muscle. Area, mean gray intensity, mode gray intensity, and max gray intensity were calculated, along with the integrated density. Measurements from Image J were divided by the ROI area to calculate the integrated density. Fold normal integrated density was calculated by comparing the normalized integrated density of the PBS-injected leg to the PS-USPION injected leg in each animal.

#### *In vivo* pharmacokinetics

EE of Alexa-fluor 647 (AF647) was characterized through UV-vis as stated in previous sections. Loaded AF647 PS-USPIONs were concentrated in PBS to 3.22 μg Fe per mL, a treatment concentration just under the solubility maximum. Three wild-type C57BL/6 mice and two homozygous βgal^−/−^ GM1-affected C57BL/6 mice (all five months old) were administered 100 μL of dye-loaded PS-USPIONs *via* tail vein injection. At 15 minutes, 1 hour, 4 hours, and 24 hours, an *In Vivo* Imaging System (IVIS, PerkinElmer IVIS Spectrum, Waltham, MA) image was taken to confirm differences in PS-USPION release profiles in diseased *versus* healthy animals, as observed in our benchtop release studies.^7^

#### Statistical analysis

Data normality was confirmed using D'Agostino and Pearson tests. Comparison between PS HD, PDI, and zeta potential was performed using unpaired Welch *t*-tests. Statistical differences in loading content under each condition were compared using Welch's one-way ANOVA test. MTS results were analyzed using multiple Welch *t*-tests. Because the integrated density data failed data normality assessments, groups were compared using multiple Mann–Whitney tests. In all cases, the alpha value was set at 0.05.

## Results

USPIONs were coated with PLA to moderate hydrophobicity and enable loading into the PLA membrane. The PLA coating was confirmed by an increase in HD from the reported 3 nm to 6.85 ± 0.35 nm. PLA-coated USPIONs (PLA-USPIONs) were then loaded into PSs by dissolution into the organic phase during PS synthesis.^6–8^ The physical parameters of each PS formulation are given in Table 1. Upon USPION loading, the PS HD increased slightly from that of unloaded HA–PLA PSs. Furthermore, the surface charge became less negative in all cases *versus* the unloaded control.

**Table 1:** USPION loading optimization parameters

PLA-USPION [μg]	HD [nm]	PDI	ZP [mV]
0	82.0 ± 10.2	0.395 ± 0.05	−24.9 ± 2.8
1	115.8 ± 17.3	0.499 ± 0.16	−9.9 ± 1.2*
5	112.0 ± 10.0*	0.329 ± 0.11	−7.3 ± 2.7**
10	109.7 ± 19.7	0.367 ± 0.10	−12.6 ± 2.1*
15	100.6 ± 13.9	0.340 ± 0.10	−13.3 ± 4.0
20	113.6 ± 21.1	0.369 ± 0.09	−12.0 ± 5.7

**Fig. 1:**
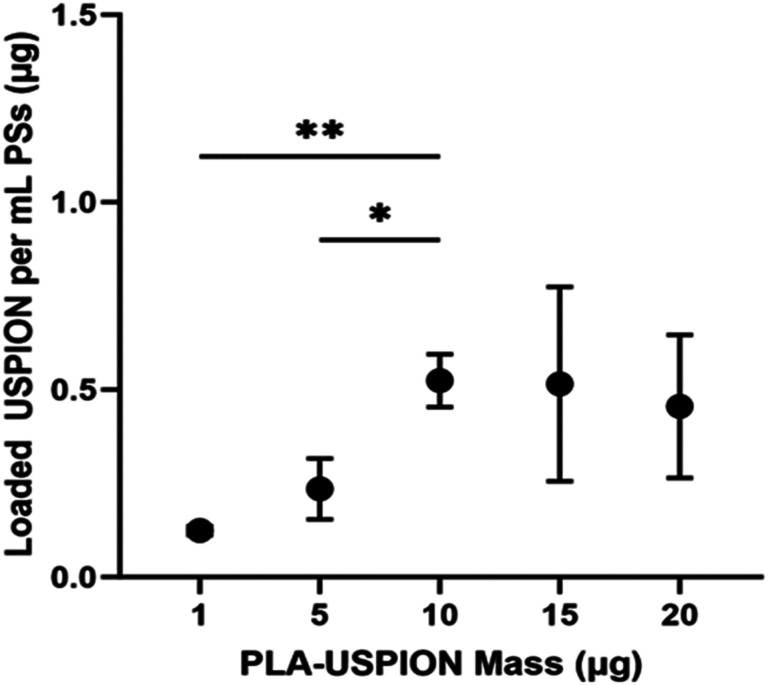
USPION mass in 1 mL of PSs (μg) plotted *versus* PLA-USPION mass (μg) used in synthesis. *p < 0.05; **p < 0.005.

USPION content in each PS sample was determined *via* ferrozine assay. The mass of USPION loaded in PSs increased with increasing PLA-USPION mass added. However, a plateau was observed following the addition of 10 μg PLA-USPION (Fig. 1), after which the deviation increased with increasing PLA-USPION content. Encapsulation efficiency, as calculated from mass content values, decreases nearly linearly with increasing USPION mass supplied. This data suggests that the PSs become saturated with USPIONs with a maximum loaded content of 0.52 ± 0.14 μg USPION/mL PS.

**Fig. 2:**
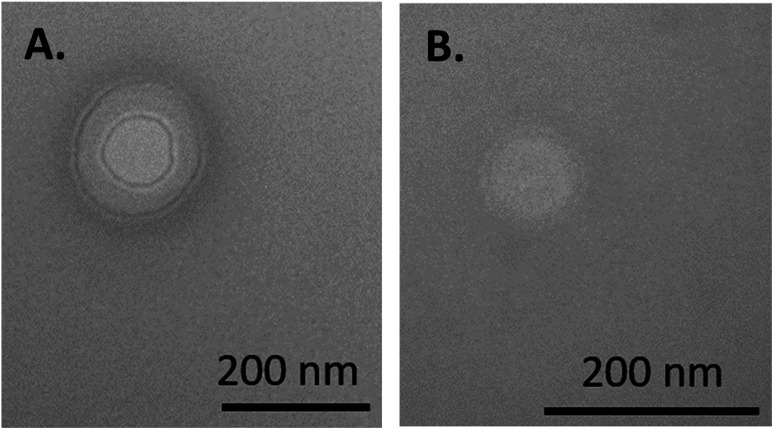
(A) PS-USPIONs and (B) No-PLA-PS-USPIONs. Samples were prepared with PTA before imaging, allowing visualization of the external soft PEG brush, while the internal dark rings are likely from USPIONs.

Transmission electron microscopy (TEM) images were taken to further confirm PS formation as well as verify membrane-localized loading (Fig. 2A). The images confirmed PS formation, with the presentation of detectable membranes, in good agreement with size and PDI measurements from DLS. USPION localization to the membrane, which was desired to leave the aqueous core available for hydrophilic drug or protein loading, was evidenced by the dark ring surrounding an otherwise light structure. TEM creates images based on the electron density of a sample, so this dark ring is assumed to be the electron-dense IO particles that have been trapped in the membrane. That dark ring is not observed in PSs without USPIONs.^7^ The most interesting observation from these images, however, was the multilamellar structure, which has not been previously observed in HA–PLA PSs to our knowledge.

**Fig. 3:**
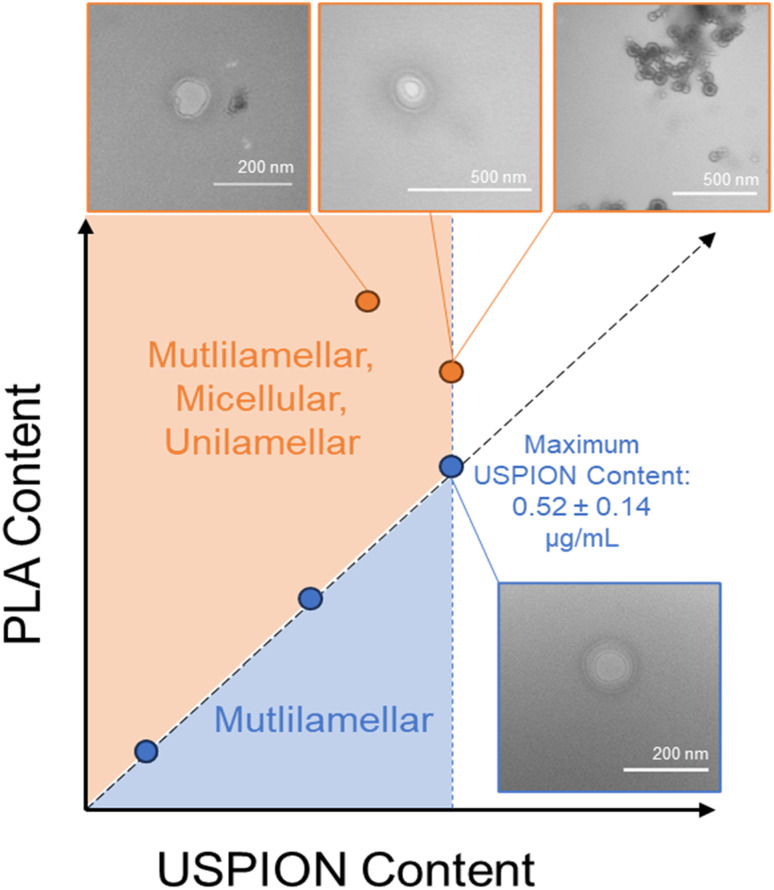
Proposed phase diagram of PSs loaded with PLA-USPIONs with corresponding TEM images. We hypothesize that the increased PLA content introduced with increasing PLA-USPIONs changes the critical packing parameter and, thus, the surface chemistry of PS self-assembly, leading to the formation of alternative structures. However, multilamellar PSs were observed at all PLA-USPION masses loaded.

All formulations examined here exhibited the multilamellar structure, although the PSs synthesized using greater PLA-USPION concentrations structurally deviated even more from the non-USPION-loaded, unilamellar PSs. The PSs synthesized with 15 and 20 μg of USPIONs exhibited a high incidence of multilamellar and unilamellar PSs as well as structures resembling an inverse micelle but with a separate, all-encapsulating outer membrane. Based on all PLA-USPION masses added to PSs and the observed overall nano-structures, we present a proposed phase diagram (Fig. 3) demonstrating the role that increasing PLA content has on the overall PS presentation, which follows expected behavior that occurs with increasing hydrophobicity.^5^

Previous studies attribute the formation of these structures to temperature or pressure differences^12,13^ but these variables were held constant in our studies. We posit that at these higher PLA-USPION levels, there is a high level of supersaturation of hydrophobic entities that pushes the critical packing parameter above 1 such that an inverted micelle is formed,^14^ which is ultimately encapsulated within a unilamellar PS to render it stable in aqueous solution. The micelle-like vesicles pack PLA-USPIONs at a high concentration within the extra hydrophobic loading space; a trade-off to this structure is a notable loss in hydrophilic loading capacity. The respective combinations of vesicular structures, each exhibiting such different loading capacities, explain the high variability seen in loaded USPION mass for these two formulations (Fig. 1).

With clinical translatability in mind, PS-USPION loaded with 10 μg of PLA-USPIONs were chosen for continued development based on uniformity in structure and reproducibility. This formulation is referred to only as PS-USPION going forward. Even so, in considering all five formulations and the consistent incidence of multilamellarity, it figures that some consistent aspect must be driving the formation of multiple membranes and we hypothesize that this aspect is the PLA coating on the USPIONs.

To investigate PLA as the driving factor for multilamellarity, we loaded the PSs with unaltered, NH_2_-activated USPIONs under the same loading protocol as before and with the same USPION concentration as the PS-USPIONs (10 μg). TEM images of the No-PLA-PSs supported membrane internalization based on the dark ring outline on a slight inset from the PS exterior, but most notably, only unilamellar morphologies were observed (Fig. 2B). The same physical characteristics were calculated with the No-PLA-PS. Additionally, co-loading capacities were evaluated with FITC-BSA as a model hydrophilic drug – an important performance parameter for this system to apply in the theranostic space. Data is presented in Table 2. Physical parameters suggested that the PSs without additional PLA assembled more consistently at a larger size, further confirming our hypothesis that increased hydrophobicity led to increased cohesion. Loading capacity was not statistically different for either USPION content or co-loading with FITC-BSA. With both formulations being largely similar, the original formulation, PS-USPIONs, was chosen for further characterization development.

**Table 2:** Comparison of PS-UPSION loading protocol with and without PLA USPION coating

	With PLA	Without PLA
HD [nm]	109.72 ± 19.69	132.1 ± 22.23*
PDI	0.367 ± 0.095	0.088 ± 0.023*
ZP [mV]	−12.6 ± 2.1	−23.8 ± 2.43*
Loaded USPIONs [μg mg^−1^ PS]	0.0198 ± 0.0051	0.0289 ± 0.0067
FITC-BSA EE [%]	36.65 ± 10.97	42.01 ± 3.84

**Fig. 4:**
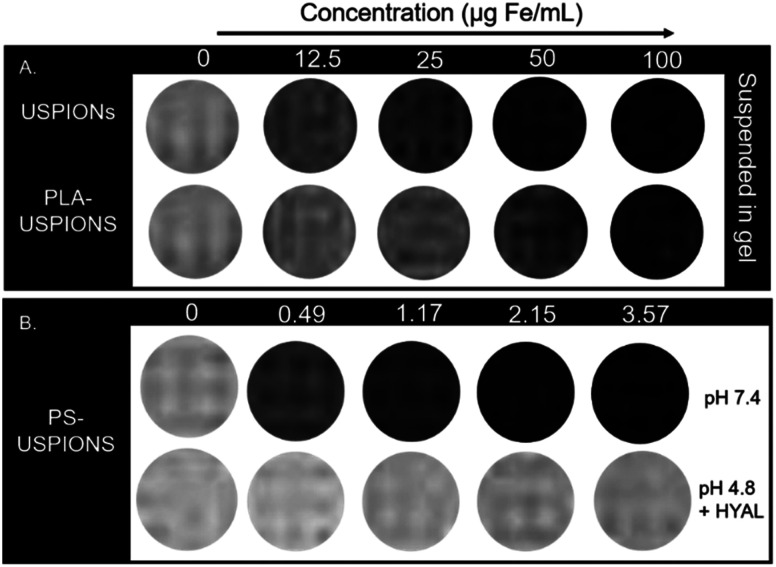
*T*_2_-Weighted MRI contrast according to concentration. (A) USPIONs and PLA-USPIONs suspended in gel at increasing concentrations. A slight decrease in contrast effect is noticeable with the addition of PLA coating. (B) PS-USPIONs suspended at multiple dilutions in salt-based buffers – PBS or Citrate buffer with HYAL for acidic. Significant loss of contrast enhancing effect observed upon incubation in disease-model environment. Appropriate control buffer references (concentration = 0 μg mL^−1^) are included with each formulation/condition set. Image still from *T*_E_ = 150 ms.

Phantom images were obtained at 3.0*T* of the USPIONs and PLA-USPIONs suspended in gel (Fig. 4A), as well as of PS-USPIONs before and after incubation in a pathological environment (1 mg mL^−1^ HYAL, pH 4.8 at 37 °C for 72 hours) to characterize MRI performance (Fig. 4B). Rapid settling of the USPIONs and PLA-USPIONs, likely due to their very small size and hydrophobicity, necessitated the use of a gelling agent. Images clearly show that our CAs were *T*_2_-oriented. It is important to note that due to the increased viscosity of the 2% agarose solutions, the relaxivity will be different than what would be expected *in situ*.^15^ The use of the gelling agent has, therefore, prevented the quantitative characterization of relaxivity. However, these images serve as a qualitative verification of contrast-altering effects. There is a slightly more pronounced darkening effect with the USPIONs prior to PLA coating, suggesting some degree of hydration blocking by the PLA, which would reduce the interaction between the CA and surrounding water protons, effectively reducing influence. However, it is difficult to compare the USPION performance before and after PLA coating without quantification, especially accounting for gel interference and potential suspension inhomogeneity, so our analysis focuses on the PS-USPIONs.

The hydrophilic exterior of the PSs allowed for direct suspension and imaging of PS-USPIONs in PBS; however, PS concentration was significantly restricted by solubility limitations. Despite this, iron concentration-dependent darkening is obvious in the PS-USPION (pH 7.4) formulation (Fig. 4B); the transverse relaxivity (*r*_2_) was calculated to be 211.14 mM^−1^ s^−1^ (Fig. S2†). Regarding stimuli-responsivity, there is a loss of darkening following the incubation of PS-USPIONs in enzymatic and acidic conditions, which matches what was observed when optimizing HA–PLA polymersomes for their release behavior in pathologic conditions.^7^ Responsivity was investigated further to determine the impact of the kinetics of HA–PLA PS degradation on corresponding *T*_2_ weighted contrast, with images taken daily for five days.

**Fig. 5:**
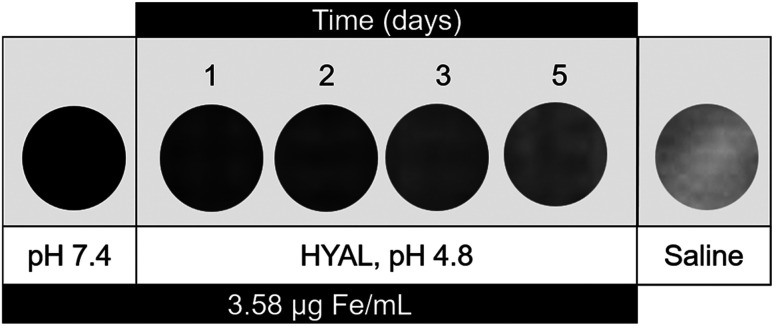
*T*_2_-weighted MRI daily response study. PS-USPIONs in neutral pH yields a consistent black contrast *versus* incubation in hyaluronidase enriched, acidic pH showed a gradual decrease in negative effect, approaching the contrast levels of a saline control. Image stills from *T*_E_ of 550 ms.

PS-USPIONs at the highest soluble concentration, corresponding to ∼3.58 μg Fe per mL, were incubated in pathologic release conditions. At all time points in the MRI time release study, an obvious difference was observable between samples suspended in neutral (pH 7.4) and acidic (pH 4.8) + enzymatic (1 mg mL^−1^ HYAL) conditions. The PBS-suspended samples maintained a constant near-black contrasting effect, similar to what was observed when calculating relaxivity (Fig. 4), while the HYAL/citrate samples were notably less dark as early as 6 hours after incubation. By hour 6, relaxivity decreased from the maximum value of 211.14 mM^−1^ s^−1^ to 26.936 mM^−1^ s^−1^, a decrease of around 87%. This lower relaxivity was maintained over the course of the study (Fig. S3†). Even so, a gradual loss of negative contrast could be observed with day-to-day direct comparisons at matched concentrations (Fig. 5).

**Fig. 6:**
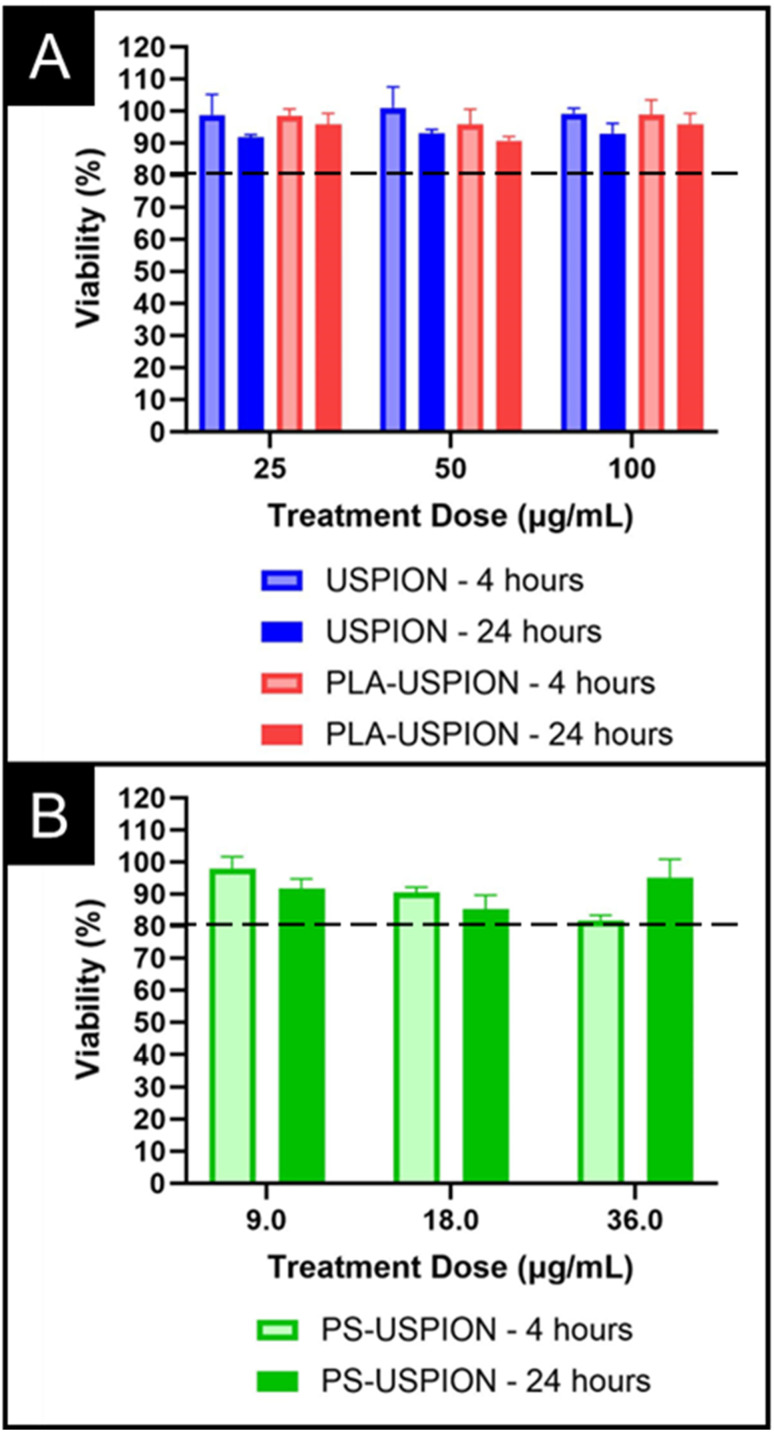
Viability analysis *via* MTS Assay over 4 and 24 hours of (A) USPION and PLA-USPION and (B) PS-USPIONs incubated.

To ensure clinical translatability, we examined the biocompatibility of PS-USPIONs *via* MTS assay. Cell survival following treatment was examined in SH-SY5Y (neuroblastoma) cells to reflect our eventual goal of functionalizing a CA for central nervous system use. Dosages up to 100 μg Fe per mL were examined. The USPIONs and PLA-USPIONs showed high biocompatibility at all treatment levels and after both 4 and 24 hours of incubation (Fig. 6), with no statistical difference observed between any dose. The PS-USPIONs showed slight but obvious dose-dependent toxicity after 4 hours, with a higher dose showing more signs of cytotoxicity, although not statistically significant. Note that high contrast is observed at iron doses as low as 3.55 μg mL^−1^ (Fig. 4), and toxicity is not observed until iron doses that are 10-fold higher. With the high strength of the PS-based CA, dosing requirements may be so low that acute inflammation conditions are never approached.

**Fig. 7:**
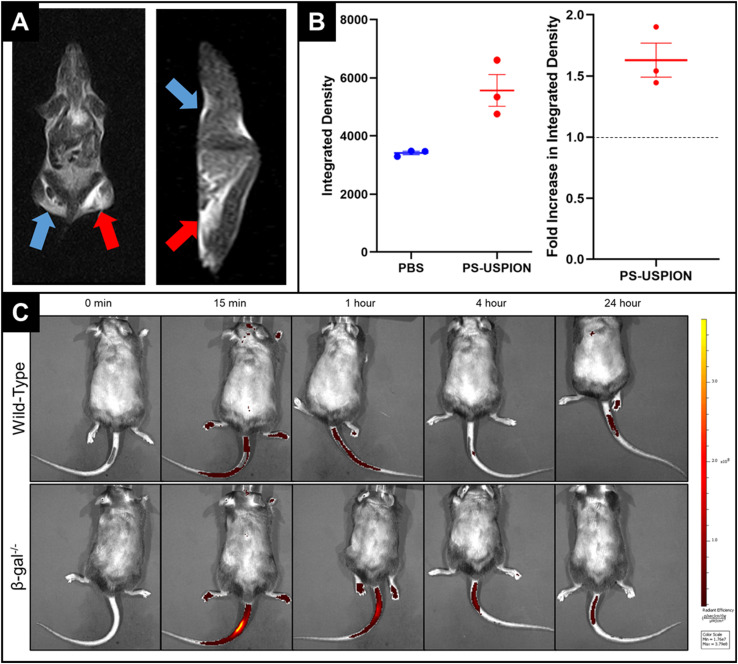
(A) Transverse view (left) and coronal view (right) of C57BL/6 mouse injected in the posterior leg muscles with either PS-USPIONs (red) or PBS (blue) taken under 0.25T MRI. The low magnet strength of 0.25*T* makes *T*_2_ weight contrast agents appear light, with a dramatic lightening apparent in the posterior muscle injected with PS-USPIONs. (B) Quantified integrated density in each posterior leg muscle taken under 0.25*T* MRI. The integrated density in both the left and right hind leg muscles were quantified using ImageJ. Measurements indicate a clear observable contrast in the PS-USPION injected leg. When normalized to the internal PBS injection, PS-USPIONs led to a fold increase in integrated density of 1.63 ± 0.24 indicating a consistent increase in contrast. (C) Representative timed IVIS images of mice following tail vein injection of PS-USPIONs. IVIS imaging indicates that PS-USPIONs are more responsive in a diseased animal, with increased release occurring in the βgal^−/−^ mouse at all observed time points. Scale bar of radiant efficiency: min = 1.76 × 10^7^, max = 3.79 × 10^8^.

*Postmortem* MRI following PS-USPION injection was then performed to evaluate the general, untargeted contrast effects in tissue. The mouse was injected with PS-USPIONs (red arrow) on the right side such that the left could be injected with PBS at the same volume (blue arrow) as an internal control. In the resulting image (Fig. 7A), *T*_2_ hyperintensity can be observed in the right leg, an obvious difference from the control leg. As will be discussed in more detail below, mice were imaged in a 0.25*T* MRI, which changes contrast presentation. This hyperintensity was quantified, with observable increases in integrated density in the PS-USPION injected leg compared to internal PBS controls (Fig. 7B). A fold increase in integrated density of 1.63 ± 0.24 was calculated, indicating the consistent *T*_2_ hyperintensity occurring across animal replicates.

Finally, pharmacokinetic behavior of PS-USPIONs was evaluated for 24 hours post injection through fluorescent monitoring *via* IVIS imaging at selected time points. WT (healthy) and βgal^−/−^ (diseased) mice were both injected with PS-USPIONs simultaneously encapsulating AF647. βgal^−/−^ mice mimic a lysosomal storage disorder called GM1 gangliosidosis^16^ which is marked by high acidity and upregulated hexosaminidase A^17^ throughout the entire body, including the brain,^18^ causing an acidic and enzymatic environment that should lyse HA–PLA PSs and therefore PS-USPIONs. IVIS images (Fig. 7C) demonstrate greater fluorescent intensity detectible in the βgal^−/−^ model mouse *versus* the WT mouse at all timepoints. Spikes in fluorescent signals were taken to indicate the release of dye from the PS, so it is clear to see that some aspect of the knock-out model mouse's physiology is driving preferential release over that of a normal mouse.

## Discussion

HA–PLA PSs are clearly capable of encapsulating and delivering USPIONs, with maximum loaded content of 0.52 ± 0.14 μg mL^−1^ of PLA-USPIONs determined (Fig. 1). This maximum loaded content is likely observed due to the membranous loading of the PLA-USPIONs by design, confirmed *via* TEM (Fig. 2A). The membrane volume of a polymersome is typically greater than its lipid analog the liposome,^19^ with thickness controlled by hydrophobic polymer molecular weight.^5^ Despite this, the volume of the polymersome membrane is still only a fraction of the overall polymersome system and significantly smaller than the interior core,^20^ leading to a maximum payload of PLA-USPIONs in this finite space. Despite the slight increase in size from unloaded controls (Table 1), the small size of these PSs with USPIONs makes them attractive for transport and diffusion in hard-to-reach structures, which could include the brain^21^ and cancerous tumors^22^ – structures for which there is a great demand for improved imaging options. Multilamellar vesicles, in general, have been shown to have higher stability than unilamellar ones, which could be beneficial for cellular delivery.^23–25^ Multilamellar structures have also been shown to not only maintain the capacity for dual hydrophobic and hydrophilic loading but do so at higher encapsulation efficiencies than unilamellar PSs.^26–29^ Additionally, release tends to be more sustained^29,30^ due to the requirement of successive membrane degradation to free all encapsulated drug. In fact, theranostic multilamellar PSs citing all of those benefits have been developed with poly(ethylene glycol)-*bl*-poly(propylene sulfide).^31^

The consistent incidence of multilamellarity suggests that the formation of multiple membranes is not PLA-USPION mass-dependent (Fig. 2A). Krack *et al*. explained the formation of multilamellar PSs upon nanoparticle loading as thermodynamically driven by a disruption to the hydrophobic/hydrophilic interface such that enclosure by multiple membranes yielded lower energy.^32^ While we agree that the hydrophobic space may be experiencing an incomplete closure from the surrounding hydrophilic cavities, especially due to the integration of USPIONs and disordered PLA chains, we also posit that the additional PLA coating abnormally alters the effective hydrophilic ratio which could be enough to alter PS morphology given that this is known to be such a crucial factor,^5^ leading to our proposed a theoretical phase diagram for this system (Fig. 3). The formation of additional membranes and aqueous core cavities could explain the size decrease observed with PLA-USPION loading when compared to uncoated USPION loading. If PLA-coated USPIONs are introduced within the large cavity of a unilamellar PS, the resulting increase in hydrophobicity may adjust the hydrogen-bonding patterns of encapsulated water making the unilamellar structure less entropically favorable by decreasing the possible hydrogen bonds that can be formed.^33^ Therefore, to minimize this loss, PS membranes may pinch inwards to form a multilamellar structure and re-maximize hydrogen bonding, which can be more easily maintained near a smaller hydrophobic region.

Additional support was provided for this hypothesis by examining the resulting loaded PS with USPIONs not coated by PLA. TEM images served as the primary source of confirmation; images portrayed generally circular vesicles with sizes in agreement with DLS, with interior dark rings representing electron density in the membrane due to IO packing. The rings were singular in these PSs, however (Fig. 2B). A thermodynamic explanation having to do with the hydrophilic fraction is likely involved, but it is most obvious to us that the phenomenon is in line with the hypothesis from Krack – the PLA-USPIONs, much bulkier than uncoated USPIONs, made it more difficult for the hydrophobic layer to become completely sealed off from hydrophilic spaces, motivating the formation of additional layers.

Physical properties, as determined by DLS, further support unilamellarity and the aforementioned hypothesis. The PSs with uncoated USPIONs were larger because they lacked the internal forces pinching the layers together to minimize unfavourable interactions. Additionally, the lower PDI indicates a more uniform self-assembly which is compatible with a simpler interaction between amphiphilic parts as allowed by the absence of additional packing of PLA strands. From this, we can tell that PLA coating was not necessary to ensure membrane localization of the USPIONs. In a comparison of the respective octanol–water partition coefficient (log *P*) of each component, the amine-activated surface is much closer to the hydrophobicity of PLA than HA, although, of course, not as close as a perfect PLA-to-PLA match with same MW. Therefore, membrane internalization is not entirely surprising but does imply that there may be a wider range of log *P* that are suited to membrane internalization for a given block copolymer, a concept that could also be worth exploring. Ultimately, the ability to co-load a therapeutic protein alongside the USPIONs, both PS-USPIONs and No-PLA-PSs were statistically indifferentiable (Table 2), making them both promising candidates for theranostic applications. With the benefits of multilamellarity in mind, we progressed to MRI performance evaluation on the PS-USPIONs.

The high relaxivity of the PS-USPION formulation suggests that encapsulation into the PS strengthened the original USPION. We hypothesize that this is because by forcing the structured aggregation of USPIONs into a particle, the PS-USPION acts as a singular particle with a much larger effective diameter. In this case, the decrease in tumbling rate would account for transverse contrast differences. It is known that increasing hydrodynamic diameter will increase *r*_2_,^34^ even just by adding dense polymer coatings, like polyethylene glycol.^35^ Because PSs are made up completely of amphiphilic block copolymer, the surface is a highly dense polymer coating of HA.

Currently, of the several IO CAs that initially received FDA approval, only Resovist remains on the market, although its use is limited to use in certain countries.^33^ It has a transverse relaxivity of 151 mM^−1^ s^−1^, but the magnetic strength under which this was measured is unclear, making direct comparison challenging. As an alternative, ferumoxytol is FDA-approved for the treatment of iron deficiency but is being explored for use as a *T*_2_ CA. Ferumoxytol has been reported to have an *r*_2_ value of 62.3 ± 3 mM^−1^ s^−1^ under similar conditions to those explored in this paper (3.0 T, saline).^36^ Therefore, compared to ferumoxytol, we have achieved a system with *T*_2_ contrast enhancement 3 times as strong as the leading alternative (Fig. 4). This high-strength *T*_2_ CA could offer the benefit of decreased dosage requirements and easier imaging discernibility.

The responsivity following PS release further strengthens this system. Within only 6 hours of incubation in the model acidic/enzymatic environment, the contrast enhancement decreased to 26.936 mM^−1^ s^−1^, less than 15% of its original value (Fig. 5). Considering our lab's characterization of HA–PLA PS release profiles,^7^ it is most likely that the contrast enhancement has decreased in the PS-USPION system following incubation in the model disease environment which leads to PS degradation. On a magnetic basis, we believe that while encapsulated into the PS, the USPIONs act collectively as one large IO nanoparticle. Upon release, the USPIONs returned to their natural strength, and because the iron content in the PS-USPIONs is lower than the 12.5 μg mL^−1^ used to image USPIONs and PLA-USPIONs (Fig. 4), and heuristically used as a minimum in many CA publications, the lower concentrations of USPIONs released from PSs are not detectible, leading to the lack of contrast. Additionally, given prior observations that unencapsulated USPIONs aggregate, it is likely that some degree of USPION settling occurred at the tube bottom, rendering them less measurable in the upper region of the tube. Either way, this change in relaxivity could then be monitored *in situ* with applicability in real-time drug tracking. If USPIONs are settling, this further substantiates our polymer degradation hypothesis and suggests facile USPION clearance following PS release *in vivo*. Until complete clearance, however, the lasting contrast enhancement could be applied to treatment monitoring or diagnostic imaging following therapeutic delivery.

Although most stimuli-responsive CAs become brighter in response to stimulus exposure, this reversal could be beneficial in a *T*_2_ CA; the CA will be very dark and easy to identify as it is moving throughout the body. Relaxivity reduction or a decrease in contrast enhancement would provide evidence of PS degradation and USPION release. With the addition of a hydrophilic drug, it would also provide evidence of successful drug delivery. Liu *et al*. developed an IO-containing PS^37^ that has similar sizing, contrast effects, and intended applications (targeting and dual drug loading) to those discussed here. These similarities lend credence to the translation of our system, especially with the success of their *in vivo* studies. While we have not achieved a CA quite as strong as theirs (611.6 mM^−1^ s^−1^), the comparative strength of our system lies in its pathologically-specific stimuli-responsivity. Our PS-USPIONs lead to changing contrast in direct response to relevant biomarkers, the enzyme- or acid-catalysed degradation of HA, as opposed to general biodegradability observed in their system. Furthermore, in their study, stimuli-responsive behavior was not confirmed *via* MRI. The multilamellar structure we observed is also unique and demonstrates a greater propensity for sustained release. It is also worth emphasizing, as Liu did, that *r*_2_ is a function of both the copolymer properties and the overall diameter and composition of USPIONs, which can vary.

The *in vitro* trends presented no cytotoxicity concerns in the USPION or PLA-USPION forms, with slight decreases in cell viability only being observed in the PS-USPION system. These results are in line with our prior observations when incubating HA–PLA nanostructures with SHSY-5Y cells.^7^ Because low MW HA causes inflammation,^38,39^ we expect a greater amount of HA–PLA PSs, which degrade into more low MW HA fragments, could result in minor cytotoxicity. Even so, the trend disappears by 24-hours, suggesting that any cytotoxicity is due to acute inflammation and is expected to be temporary. However, *in vivo* clearance studies as well as chronic dosing exposure would need to be performed to confirm elimination and biocompatibility, focusing on cytokines and anti-PEG antibodies produced over time and dose.

*Postmortem* imaging exposed a clear contrast effect observable in PS-USPION injected *vs*. PBS injected muscles, supporting the translatability of PS-USPIONs in tissue systems. The elicited effect of the CA under 0.25*T* MRI was to brighten *versus* darken, which was observed under 3*T* MRI, as is clearly shown on all phantom images. The reversal of influence is due to the extremely low strength of the MRI at only 0.25 T. It has been shown that IO nanoparticles can become positive *T*_2_ agents when imaged under low-field MRI,^40^ as is the case here. Therefore, these preliminary images confirm that our system generates a readable difference in tissue even on a weaker MRI, supporting its general strength in altering relaxivity. This increase in contrast was also measurable, with a 1.63 ± 0.24-fold increase in measurable integrated density when comparing PS-USPION-injected legs to PBS-injected internal controls on the same animal. It is promising that this increase in contrast enhancement is highly repeatable *in vivo*, demonstrated by a low animal-to-animal deviation. Additional animal imaging at higher magnetic strength (3.0 T or 7.0 T) will be required to gauge clinical effects more realistically.

Pharmacokinetics were examined by synchronized IVIS imaging in both healthy (Wild-Type (WT)) and GM1-affected (βgal^−/−^) mice to assess release of AF-647 from PS-USPIONs after dose-matched tail vein injections (Fig. 7C). Previously, fluorescein (FITC), encapsulation by PS has been shown to quench fluorescent signals;^41^ accordingly, an increase in detectable fluorescent signal could then be reliably used to quantify payload release from PSs. Based on our previous studies,^42^ we expect to see the same quenching effect when our loaded dye, AF-647, is encapsulated in PS-USPIONS. Therefore, we monitored mice post-injection to identify increases in fluorescence over time to indicate *in vivo* release profiles. We expected to see increased fluorescent release near the injection site in βgal^−/−^ mice, which model GM1 gangliosidosis, leading to an acidic and enzymatic environment^43–45^ that should lyse HA–PLA PSs and, therefore, PS-USPIONs. The greater radiant efficiency in the GM1-affected mouse tail compared to WT at each time point after tail vein injection suggests preferential PS degradation with disease as the fluorescent signal from free AF-647 becomes detectible. Assuming differences in release are due to differences in the circulatory microenvironment, which is reasonable given the nonspecific administration route and lack of targeting ligands, the greater payload release is likely a result of elevated Hexosaminidase A levels in blood serum in GM1-affected mice.^6^ This observance lends strong *in vivo* support for the PS-USPIONs’ performance as a responsive release system, which mimics what was observed in our benchtop release studies performed when optimizing HA–PLA PSs for protein delivery.^7^ It is encouraging that the *in vivo* performance of our system matches release profiles expected in pathologic acidic and enzymatic conditions. To increase applicability as a theranostic tool, we could identify time points that tend to represent the greatest PS degradation/payload release to establish an optimum window for real-time MRI monitoring in clinical applications. For this, it would be helpful to establish the time of absolute clearance of the PSs. Our previous work indicates that PSs are cleared at different rates depending on injection routes and targeting ligands.^42^

## Conclusions

We have created a biocompatible, responsive CA-PS system based on USPIONs and HA–PLA PSs. Polymer-matched USPION coating based on the hydrophilic block of the PS copolymer, PLA, was successfully added to ensure the membranous encapsulation of these USPIONs. Using log *P* matching, have not only left the PS open for co-loading of a model protein, but we have also created a multilamellar vesicle system that will allow for sustained release for continued MRI monitoring, possibly facilitating decreased administration frequency. Through the tight packing into the PS structure, effectively forcing the nanoparticles to act as a single large aggregate, the contrast enhancement observed using PS-USPION is 3-fold greater than the most promising clinical candidate, ferumoxytol. Preliminary *in vivo* and *ex vivo* studies in GM1-affected mice and environments confirmed contrast strength and preferential release towards enzymatic, acidic microenvironments. The ultimate result is a targetable, theranostic system that can be tracked until the time of delivery to pathological sites, after which carrier degradation triggers a measurable relaxivity change.

## Author contributions

Dorian Foster – conceptualization, data curation, formal analysis, investigation, methodology, validation, visualization, writing – original draft, writing – review & editing; Naisha Shah – investigation, methodology, validation; Alaura Cakley – investigation, methodology, validation; Ronald Beyers – methodology, validation, visualization; Jessica Larsen – conceptualization, data curation, formal analysis, funding acquisition, project administration, resources, supervision, validation, visualization, writing – review & editing.

## Data availability

The data supporting this article have been included as part of the ESI.† Any additional data requests should be made by contacting the corresponding author.

## Conflicts of interest

There are no conflicts to declare.

## Supplementary Material

BM-013-D4BM01583E-s001
