# Supplementary material for: Multilamellar hyaluronic acid-b-poly(lactic acid) polymersomes for pathology-responsive MRI enhancement
Source: Biomater Sci. 2025 Apr 17;13(11):2961–72. doi: 10.1039/d4bm01583e (PMC12019155; doi:10.1039/d4bm01583e)
Supplement: BM-013-D4BM01583E-s001 [file BM-013-D4BM01583E-s001.pdf]

Supplementary Information (SI) for Biomaterials Science.
This journal is © The Royal Society of Chemistry 2025

Multilamellar Hyaluronic Acid-B-Poly(Lactic Acid) Polymersomes For Pathology-Responsive MRI Enhancement

Dorian Foster*[a], Naisha Shah[b], Alaura Cakley[a], Ronald Beyers[c], and Jessica Larsen[a,d]

Supplemental Figures

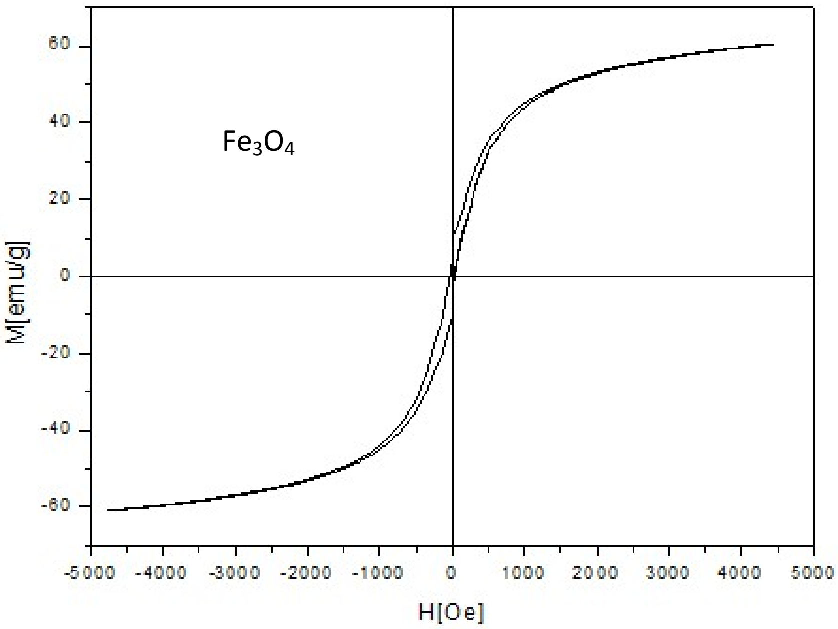

Figure S1: M-H plot for Amine functionalized USPIONs. Provided by the supplier (US Research Nanomaterials) and measured at room temperature

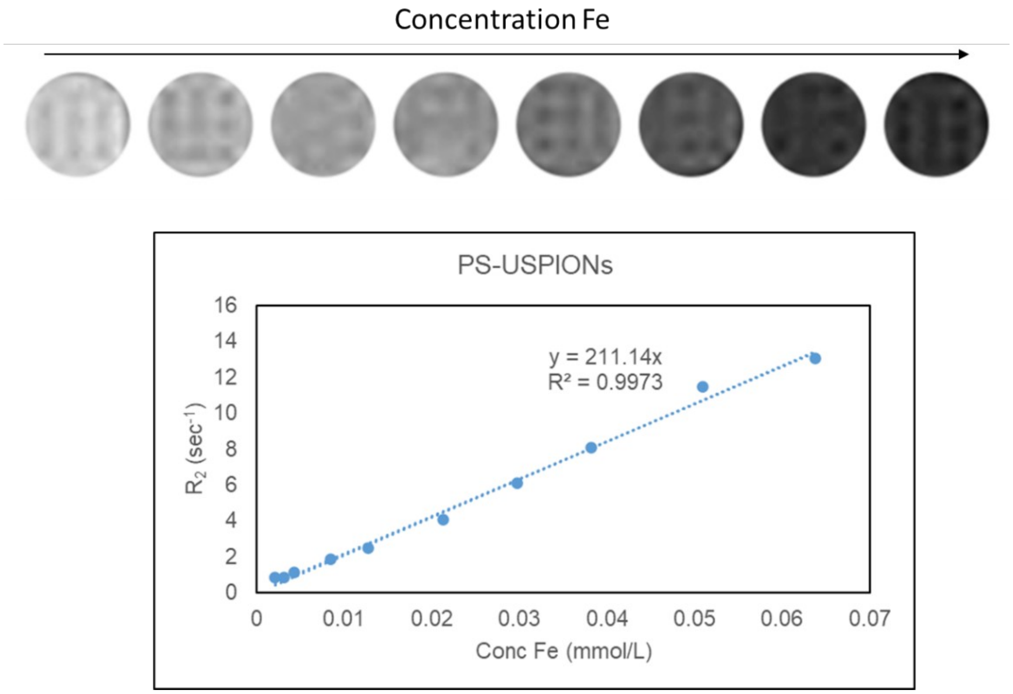

Figure S2: Relaxivity plot for PS-USPIONs. Plot of R_2 (or $1/T_2$) versus concentration of iron and corresponding linear fit, where slope is equal to r_2. R2 was calculated through a Matlab program set up for automatic calculations on given regions of interest. The images used for calculation were taken at Auburn University on a 3.0 T MRI scanner.

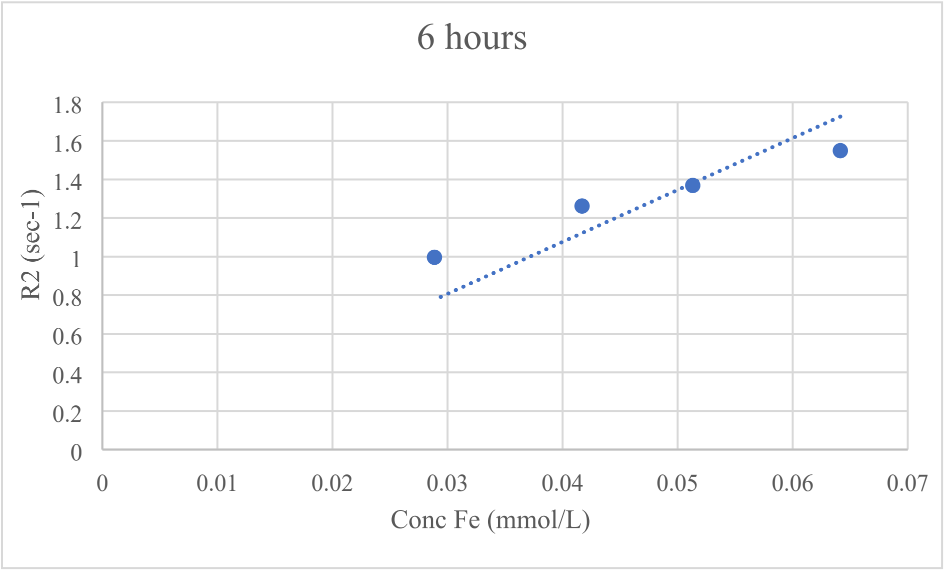

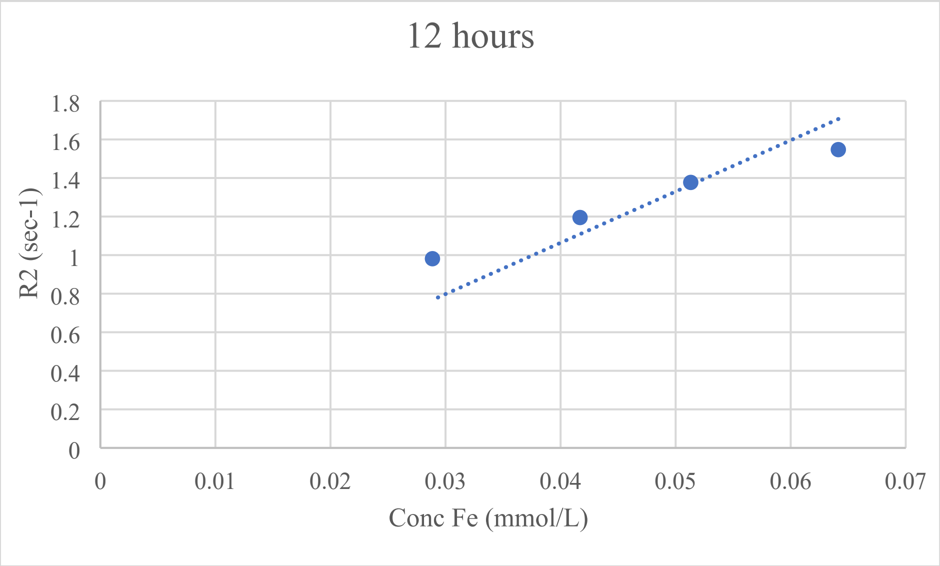

Figure S3: Relaxivity plot for PS-USPIONs after Pathologic Buffer Incubation over Time . Plot of R_2 (or $1/T_2$) versus concentration of iron and corresponding linear fit, where slope is equal to r_2. R2 was calculated through a Matlab program set up for automatic calculations on given regions of interest. The images used for calculation were taken at Auburn University on a 3.0 T MRI scanner. Each timepoint was used to calculate relaxivity with a linear plot going through (0,0).

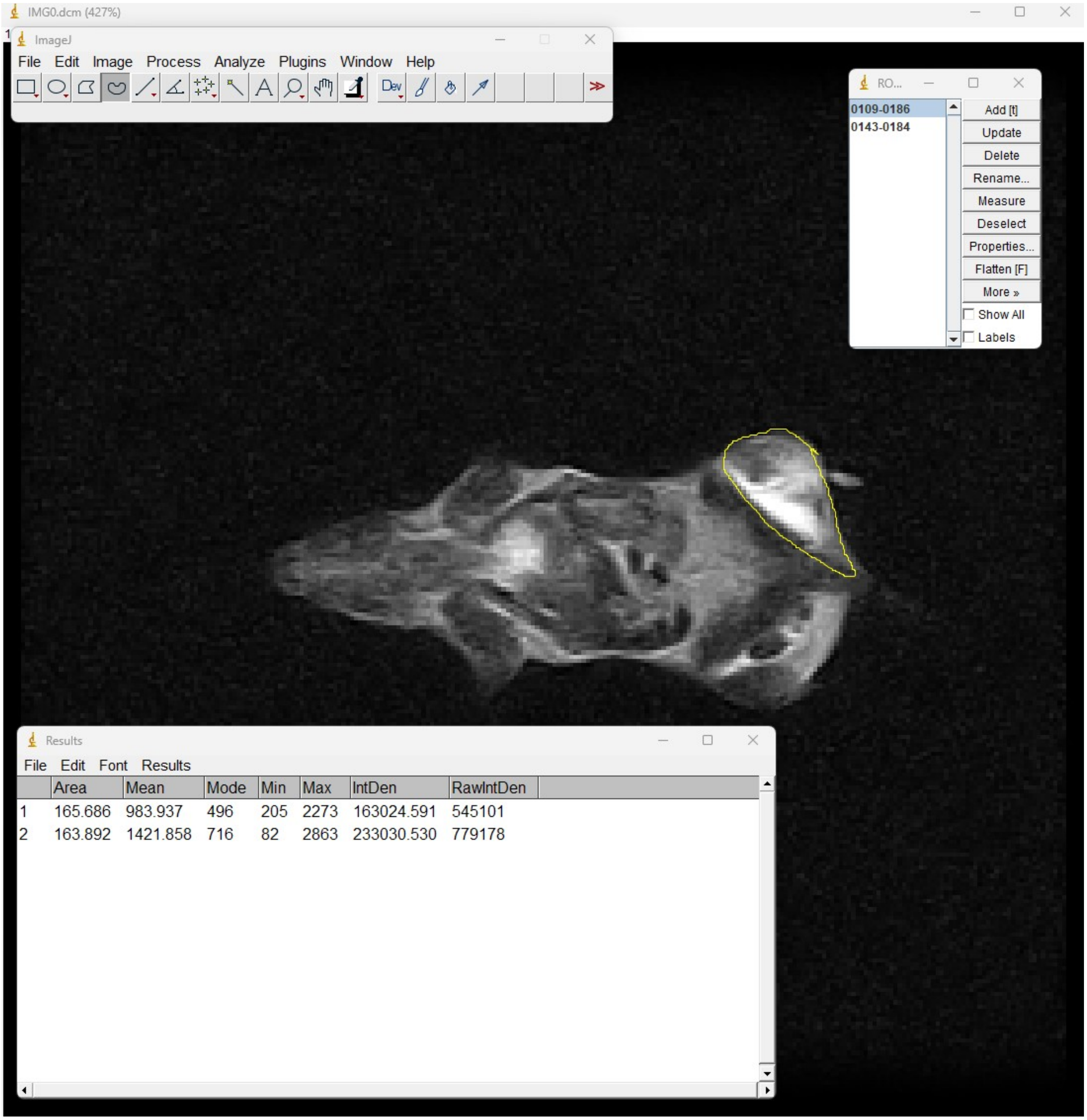

Figure S4: ImageJ Analysis of Integrated Density. Regions of interest (ROIs) were drawn around each leg muscle. Area, Mean Gray Intensity, Mode Gray Intensity, and Max Gray Intensity were calculated, along with the Integrated Density. To calculate the integrated density as reported in Figure 7B, measurements from Image J were divided by the ROI area. Fold Normal Integrated Density was calculated by comparing the normalized integrated density of the PBS-injected leg to the PS-USPION injected leg in each animal.
